# Metabolic Profiles Point Out Metabolic Pathways Pivotal in Two Glioblastoma (GBM) Cell Lines, U251 and U-87MG

**DOI:** 10.3390/biomedicines11072041

**Published:** 2023-07-20

**Authors:** Filipa Martins, David van der Kellen, Luís G. Gonçalves, Jacinta Serpa

**Affiliations:** 1 iNOVA4Health, NOVA Medical School|Faculdade de Ciências Médicas, Universidade NOVA de Lisboa, Campo dos Mártires da Pátria, 130, 1169-056 Lisboa, Portugal; filipa.martins@nms.unl.pt (F.M.); van-derkellen@hotmail.com (D.v.d.K.); 2 Instituto Português de Oncologia de Lisboa Francisco Gentil (IPOLFG), Rua Prof Lima Basto, 1099-023 Lisboa, Portugal; 3 Instituto de Tecnologia Química e Tecnológica (ITQB) António Xavier da Universidade Nova de Lisboa, Av. da República, 2780-157 Oeiras, Portugal; lgafeira@itqb.unl.pt

**Keywords:** glioblastoma, cancer metabolism, metabolic remodeling, metabolomics, gene expression profiles

## Abstract

Glioblastoma (GBM) is the most lethal central nervous system (CNS) tumor, mainly due to its high heterogeneity, invasiveness, and proliferation rate. These tumors remain a therapeutic challenge, and there are still some gaps in the GBM biology literature. Despite the significant amount of knowledge produced by research on cancer metabolism, its implementation in cancer treatment has been limited. In this study, we explored transcriptomics data from the TCGA database to provide new insights for future definition of metabolism-related patterns useful for clinical applications. Moreover, we investigated the impact of key metabolites (glucose, lactate, glutamine, and glutamate) in the gene expression and metabolic profile of two GBM cell lines, U251 and U-87MG, together with the impact of these organic compounds on malignancy cell features. GBM cell lines were able to adapt to the exposure to each tested organic compound. Both cell lines fulfilled glycolysis in the presence of glucose and were able to produce and consume lactate. Glutamine dependency was also highlighted, and glutamine and glutamate availability favored biosynthesis observed by the increase in the expression of genes involved in fatty acid (FA) synthesis. These findings are relevant and point out metabolic pathways to be targeted in GBM and also reinforce that patients’ metabolic profiling can be useful in terms of personalized medicine.

## 1. Introduction

Gliomas are the most common primary brain tumors, accounting for 24% of all central nervous system (CNS) tumors [1]. The broad category of gliomas includes glioblastomas (GBMs), high-grade tumors (WHO grade IV) that account for 14.2% of all CNS tumors and 50.1% of all malignant CNS tumors [1]. GBM are highly lethal, mainly due to their inaccessible localization in the brain, high proliferation rate, increased heterogeneity, and infiltrative/invasive capacity [2]. According to the current glioma classification (WHO 2021), GBMs correspond to *IDH*-wild-type diffuse and astrocytic gliomas that present at least one of the following features: microvascular proliferation, *TERT* promoter mutation, *EGFR* gene amplification or 7+/10− chromosome copy number changes [3]. The previous GBM classification included a broader group of tumors, such as *IDH*-mutant gliomas, which corresponded to a median overall survival of 15 months [4,5]. With the new classification, this narrower group includes only the most aggressive tumors, having a median overall survival of 8 months, according to the latest statistical reports [1,6]. The current clinical treatment involves a multidisciplinary approach, consisting of maximal tumor resection, followed by adjuvant radiotherapy and chemotherapy (temozolomide, TMZ) [7]. However, complete surgical removal is not possible because of the high invasiveness degree, leading to later disease progression or recurrence [8]. This has led to the search for new biomarkers that could act as predictors of patient outcomes or treatment responses. 

In the last years, cancer metabolism has acquired a central position in oncology research, since metabolic adaptation is crucial to sustaining tumor growth [9]. In the case of malignant gliomas, the glutaminergic cycle suffers a remodeling through the abrogation of glutamine synthetase [10,11] and overexpression of glutamine transporters ASCT2 [12] and SNAT3 [13]. Thus, these neoplasms present a phenotype of high glutamine dependence, since they are unable to produce it, acting as ‘glutamine traps’ by importing glutamine from the tumor microenvironment [2,14]. Nevertheless, these tumors are able to fully metabolize glutamine, since they express glutaminase isoform 1 (GLS-1) [15], using this amino acid as a carbon (C) and nitrogen (N) source [9,16,17]. Furthermore, GBM cells often present increased glycolysis, including overexpression of glucose transporters GLUT1 and GLUT3 [18,19,20] and lactate transporters MCT1 and MCT4 [21,22,23]. These adaptations may reflect in the use of glucose for biosynthesis, while glutamine is used for bioenergetics, acting as a glucose substitute [9].

Even though the amount of knowledge generated by cancer metabolism research is increasing, its application to cancer therapy has been scarce. With this article, we want to take advantage of the knowledge obtained from metabolic profiling, providing insights for future definition of metabolic profiles useful for clinical applications. Here, we investigated the metabolic and gene expression profiles of U251 and U-87MG GBM cell lines when exposed to glucose, glutamine, glutamate, and lactate, and the impact of these metabolites on cell features crucial for cancer progression, such as proliferation, migration, and viability. Unraveling the metabolome with a sensitive and high-throughput technique such as nuclear magnetic resonance (NMR) in combination with gene expression profiling with qPCR allowed for the identification of metabolite variations and altered pathways, providing insights into GBM biology and metabolic adaptive capacity.

## 2. Materials and Methods

### 2.1. GBM Patient Data Source

Data for GBM were extracted from the FireBrowse database (http://www.firebrowse.org, accessed on 25 May 2023). The mRNA expression of several genes involved in GBM metabolism in patients was explored in the GBM cohort of The Cancer Genome Atlas (TCGA). Tumors were classified according to 2016 WHO CNS tumor classification [4], which still includes *IDH1* mutant high-grade gliomas as GBM. *p*-value lower than 0.05 denoted statistical significance. 

### 2.2. Cell Culture

Two commercial GBM cell lines, U-87MG (HTB-14, American Type Culture Collection (ATCC)) and U251 (09063001, European Collection of Authenticated Cell Cultures (ECACC)), were used and cultured in Dulbecco’s modified eagle medium F-12 (DMEM/F-12; 11330-032, Gibco, Life Technologies, New York, NY, USA), supplemented with 10% fetal bovine serum (FBS; P40-37500, PAN Biotech), 1% antibiotic–antimycotic (AA; P06-07300, PAN Biotech, Aidenbach, Germany), and 50 µg/mL gentamicin (15750-060, Gibco, Life Technologies). All cells were maintained in a humidified environment of 5% CO_2_ at 37 °C. Cells were cultured until an optical confluence of 75–100% and then detached with 0.05% trypsin-EDTA 1× (25300-054, Invitrogen, Thermo Fisher Scientific, New York, NY, USA) at 37 °C for approximately 5 min, and split to new plates according to the experimental procedures.

Before in vitro assays, cells were washed with phosphate buffer saline (PBS) 1× and synchronized under starvation (culture medium without FBS) overnight. During exposure to experimental conditions (24 h), cells were maintained in DMEM/F-12 without L-glutamine, HEPES, and glucose (L0091-500, Biowest, Nuaillé, France), with 1% FBS. Experimental conditions comprised 5 mM glucose (G8270, Sigma-Aldrich, New York, NY, USA), 6 mM L-glutamine (25030-024, Gibco), 6 mM glutamate (6382-01-0, Sigma Aldrich), and 10 mM sodium lactate (NaLac; 1.06522.2500, Merck, New York, NY, USA).

### 2.3. Nuclear Magnetic Resonance (NMR) Spectroscopy

Cells (2.6 × 10^7^ cells/flask) were seeded in 175 cm^2^ culture flasks. After exposure to experimental conditions, cells were harvested with PBS 1× (washed twice), scraped, and centrifuged at 155× *g* for 10 min. Methanol and chloroform extraction was performed to separate organic and aqueous phases. Cold methanol was added to the cell pellets (4 mL methanol/1 g cell pellet), followed by water (twice the volume of methanol). After a 5 min incubation on ice, chloroform (1 vol) was added to the mixture, followed by water (1 vol). The samples were incubated for 10 min on ice and centrifuged at 1700× *g* for 15 min at 4 °C. Both organic and aqueous phases were collected with a glass pipette and stored at −20 °C until sample analysis. Chloroform extracts (organic phase, containing insoluble water compounds) and methanol/water extracts (aqueous phase, containing water-soluble compounds) were analyzed using ^1^H-NMR spectroscopy. Lyophilization of both extracts was carried out using a SpeedVac Plus system and then organic samples were dissolved in deuterated chloroform (CD_3_Cl) and aqueous sample in deuterated water (D_2_O) with 0.16 mM 3-(trimethylsilyl)propionic-2,2,3,3-d4 acid (TSP), as chemical shift reference, and 82 mM potassium phosphate buffer (KPi pH 7.4). ^1^H-NMR spectra of aqueous samples were obtained at 25 °C in a magnetic field of 800 MHz using an UltrashieldTM 800 Plus Spectrometer (Bruker, Billerica, MA, USA) with a TCI cryoprobe, using *noesygppr1d* pulse program. Organic sample spectra were acquired in a 500 MHz magnetic field in the 500 UltraShieldTM Spectrometer (Bruker) using a 5 mm TCI-z Prodigy cryoprobe (5 mm), using *zg* pulse program. TopSpin 4.0.7 software (Bruker) was used for spectra acquisition and processing. Compound identification on aqueous samples’ spectra was made by resorting to the Human Metabolome Database (HMDB; http://www.hmdb.ca/, accessed on 24 April 2023), and Chenomx NMR Suite software version 8.1 (Chenomx Inc., Edmonton, Canada), which was also used for metabolite quantification. Organic spectra were processed using NMRProcFlow 1.4 software (nmrprocflow.org, accessed on 1 June 2023), and bucket assignment to each functional group or lipidic constituent was performed based on Amiel et al. 2019 [24]. Data analysis was performed using the MetaboAnalyst 5.0 platform (https://www.metaboanalyst.ca/, accessed on 15 May 2023).

### 2.4. Reverse Transcription and Quantitative PCR (RT-qPCR)

Cells were seeded in 6-well plates (4 × 10^5^ cells/well) and exposed to culture conditions. Total RNA was extracted using the RNeasy Mini Extraction kit (74104, Qiagen, Venlo, The Netherlands), according to the manufacturer’s protocol. cDNA was synthesized from 1 µg RNA using SuperScript II Reverse Transcriptase (18080e44, Invitrogen, New York, NY, USA), according to the manufacturer’s protocol. Relative quantification using quantitative PCR (qPCR) was performed using SYBR Green PCR Master Mix (04707516001, Roche, Basel, Switzerland), according to the manufacturer’s protocol. Real-time PCR was carried out using a Lightcycler^®^ 480 System instrument (05015243001, Roche). The primers used are presented in Table 1.

### 2.5. Cell Death Analysis Using Flow Cytometry

To analyze the effects of the experimental conditions on cell viability, GBM cell lines were seeded in 24-well plates (1 × 10^5^ cells/well). After a 24 h exposure to experimental conditions, the supernatant was collected, and adherent cells were detached with 0.05% trypsin-EDTA. Cells and supernatant were collected in the same tube and centrifuged at 155× *g* for 3 min. Then, cell pellets were incubated with 0.5 µL annexin V-fluorescein isothiocyanate (annexin V-FITC; 640906, BioLegend, San Diego, CA, USA), in annexin V binding buffer 1× (10 mM HEPES (pH 7.4; 391333, Millipore, New York, NY, USA), 140 mM sodium chloride (NaCl; 106404, Merck), 2.5 mM calcium chloride (CaCl_2_; 449709, Sigma-Aldrich) for 15 min in the dark at room temperature. Cells were resuspended in 200 µL PBS 1×/0.1% (*v*/*w*) bovine serum albumin (BSA; A9647, Sigma-Aldrich) and centrifuged at 155× *g* for 2 min. The remaining pellets were resuspended in annexin V binding buffer 1× and 2.5 µL of 50 µg/mL propidium iodide (PI; P4170, Sigma-Aldrich). Samples were analyzed using flow cytometry in a FACScalibur (Becton Dickinson, Franklin Lakes, NJ, USA) and data were treated using FlowJo X 10.0.7 (http://flowjo.com, accessed on 5 June 2023) software.

### 2.6. Cell Proliferation Assay

Cells were seeded in 24-well plates (5 × 10^4^ cells/well) and, after being exposed to experimental conditions, were collected at 0, 6, 12, 24, 32, and 48 h. The cell number per mL was calculated using a Bürker counting chamber and cell viability was determined using 0.4% (*w*/*v*) trypan blue stain (15250-061, Gibco) at a ratio of 1:5. Three replicates were analyzed for each cell line and experimental condition.

### 2.7. Wound Healing Assay

Cells were seeded in 24-well plates (1 × 10^5^ cells/well). At 90% of confluency, cells incubated for 3 h with 5 μg/mL mitomycin-C (M4287, Sigma) to inhibit cell proliferation. A linear scratch across the well diameter was made with a P200 pipette tip, the media were replaced to remove debris and cells in suspension and the experimental conditions were applied. Bright field images of each well were acquired using an Olympus IX53 Inverted Microscope until 48 h. The wound closure was quantified using ImageJ software, version Java 8 (imagej.nih.gov/ij/, accessed on 10 June 2023).

### 2.8. Statistical Analysis

For patients’ mRNA seq data analysis, one-way ANOVA was used to compare the three groups, with the nonparametric Kruskal–Wallis test, since the populations did not have a normal distribution (Table S1). Differences were determined statistically significant at *p*-value < 0.05.

MetaboAnalyst 5.0 (http://www.metaboanalyst.ca/, accessed on 25 May 2023), online platform was used for NMR bioinformatic analyses. This software was used for both multivariate and univariate statistical analyses. Samples were normalized by sample weight and, for the multivariate analysis, the metabolite concentrations were scaled by autoscaling. Multivariate analysis involved principal component analysis (PCA), an unsupervised method to assess variance between samples and their clustering. PCA, heatmaps, and *t*-test plots were generated using the same platform.

For the other experiments, all data were analyzed using Student’s *t*-test, one-way ANOVA, or two-way ANOVA in GraphPad Prism v9 software (www.graphpad.com/, accessed on 13 June 2023). The assays were performed with at least 3 biological replicates per condition and the differences were determined statistically significant at *p*-value < 0.05.

## 3. Results

### 3.1. The Expression of Metabolic Key Genes Is Altered in Glioblastoma (GBM) Specimens

**Figure 1:**
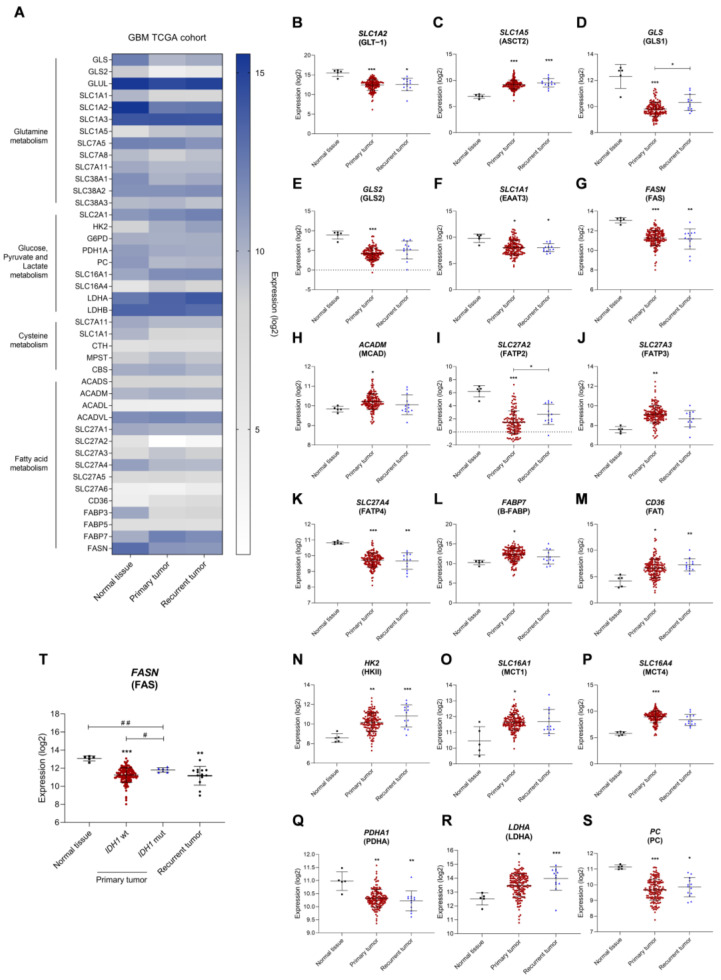
Distinct gene expression profiles between glioblastoma (GBM) and normal tissue indicate the metabolic remodeling occurring in GBM. (**A**) Heatmap of metabolism-related genes in normal tissue, primary tumor, and recurrent tumor of GBM TCGA cohort. Different expression levels in the primary tumor: decreased glutamate transporter *SLC1A2* (**B**), increased glutamine transporter *SLC1A5* (**C**), decreased glutaminase *GLS* (**D**) and *GLS2* (**E**), decreased glutamate and cysteine transporter *SLC1A1* (**F**), decreased fatty acid synthase *FASN* (**G**), increased medium-chain acyl-coenzyme A dehydrogenase *ACADM* (**H**), decreased fatty acid transporter 2 *SLC27A2* (**I**), increased fatty acid transporter 3 *SLC27A3* (**J**), decreased fatty acid transporter 4 *SLC27A4* (**K**), decreased brain fatty acid-binding protein *FABP7* (**L**), increased fatty acid translocase CD36 (**M**), increased hexokinase II *HK2* (**N**), increased monocarboxylate transporters *SLC16A1* (**O**) and *SLC16A4* (**P**), decreased pyruvate dehydrogenase *PDHA* (**Q**), increased lactate dehydrogenase A *LDHA* (**R**) and decreased pyruvate carboxylase *PC* (**S**). Fatty acid synthase expression according to *IDH1* mutational status (**T**). All data are represented as mean ± SD. * *p* < 0.05, ** *p* < 0.01, *** *p* < 0.001, Kruskal-Wallis test. # *p* < 0.05, ## *p* < 0.01, Mann-Whitney test.

To understand how GBM affects the expression of several genes involved in important metabolic pathways, mRNA seq data from 171 patients’ samples was extracted from the GBM TCGA cohort. These corresponded to three sample types: normal tissue (5 samples), primary tumor (153 samples), and recurrent tumor (13 samples). These GBM samples were classified according to the 2016 WHO CNS tumor classification [4]. A different gene expression profile was observed when comparing normal tissue with primary and recurrent tumors (Figure 1A). Genes related to glutamine and glutamate transport were differently expressed: decreased glutamate transporter GLT-1 gene (*SLC1A2*) (Figure 1B), increased *SLC1A5* gene encoding the glutamine transporter ASCT2 (Figure 1C), decreased *GLS* and *GLS2* genes encoding for two glutaminase isoforms (Figure 1D,E, respectively), and decreased *SLC1A1* gene encoding the glutamate and cysteine transporter EAAT3 (Figure 1F) in primary tumor. Regarding fatty acid metabolism, there was decreased fatty acid synthase gene *FASN* (Figure 1G); increased *ACADM* gene encoding the medium-chain acyl-coenzyme A dehydrogenase (Figure 1H); decreased *SLC27A2* gene encoding fatty acid transporter 2, FATP2 (Figure 1I); increased *SLC27A3* gene encoding the fatty acid transporter 3, FATP3 (Figure 1J); decreased *SLC27A4* gene encoding the fatty acid transporter 4, FATP4 (Figure 1K); increased *FABP7* gene encoding brain fatty acid-binding protein B-FABP (Figure 1L); and increased *CD36* gene encoding the fatty acid translocase FAT (Figure 1M). Regarding glycolysis, we observed increased hexokinase II gene, *HK2* (Figure 1G); increased monocarboxylate transporter genes, *SLC16A1* encoding MCT1 (Figure 1H) and *SLC16A4* encoding MCT4 (Figure 1I); decreased pyruvate dehydrogenase gene, *PDHA* (Figure 1J); increased lactate dehydrogenase A gene, *LDHA* (Figure 1K); and decreased pyruvate carboxylase gene, *PC* (Figure 1S). Considering the *IDH1* mutational status, a significant increase in *FASN* expression in *IDH1* mutated primary tumors was observed compared to *IDH1* wild-type (wt) tumors (Figure 1T). The expression of other genes associated with these pathways can be found in Supplementary Figure S1.

### 3.2. Glucose, Lactate, Glutamine, and Glutamate Bioavailability Impacts the Expression Profile of Metabolic Key Genes in GBM Cell Lines Differently

**Figure 2:**
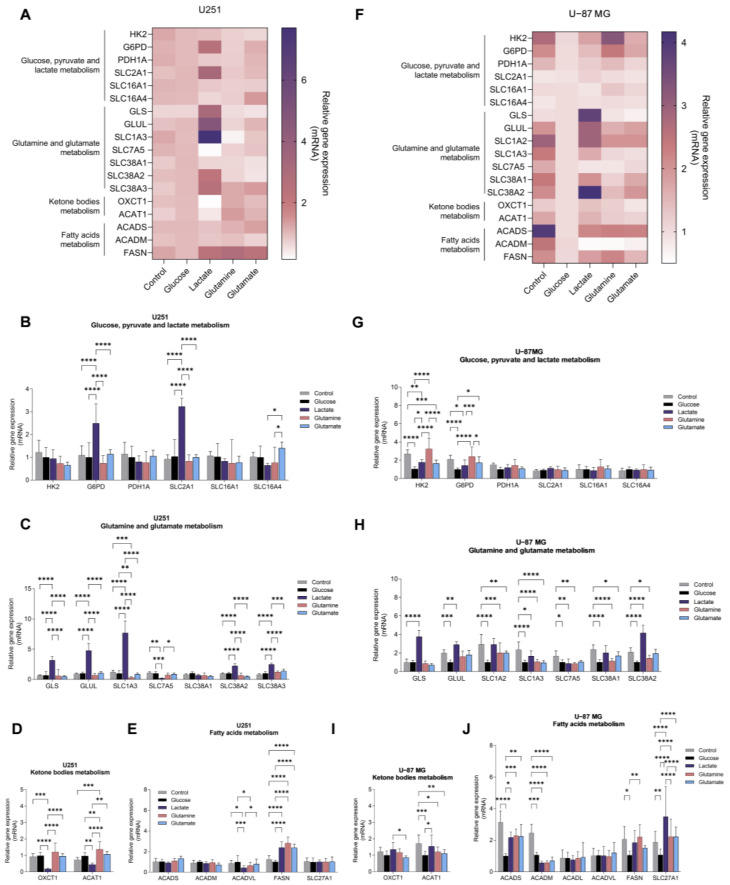
GBM cell lines show different gene expression profiles in the presence of key metabolites: glucose, glutamine, glutamate and lactate. U251 (**A**–**E**) and U-87MG (**F**–**J**) cell lines present distinct gene expression profiles according to the metabolite to which they were exposed. Genes from several metabolic pathways were assessed: glucose, pyruvate, and lactate metabolism (**B**,**G**); glutamine and glutamate metabolism (**C**,**H**); ketone body metabolism (**D**,**I**); and fatty acid metabolism (**E**,**J**). All data were normalized to the glucose sample and represented as mean ± SD. * *p* < 0.05, ** *p* < 0.01, *** *p* < 0.001, **** *p* < 0.0001.

Since glucose- and glutamine-related pathways are affected in primary GBM, we investigated the impact of glucose, lactate, glutamine, and glutamate as carbon sources in the expression of metabolic key genes in the two GBM cell lines. Control conditions were considered cell-cultured without supplementation of glucose, lactate, glutamine, and glutamate. We assessed the expression of genes related to glucose-dependent pathways (glycolysis, pentose phosphate pathway (PPP), and gluconeogenesis), glutamine and glutamate metabolism and transport, lipid metabolism (FA synthesis, β-oxidation, and ketone body production), and transport. GBM cell lines displayed different expression profiles of metabolic genes in response to the exposure to each carbon source (Figure 2A,F). 

U251 cell line exposed to glucose presented a similar expression profile when compared to the control condition (in the absence of glucose and glutamine for 24 h) (Figure 2B–E), while U-87MG cells decreased the expression of genes encoding glycolytic enzymes (*HK2* and tendency to decrease *PDH1A*) and the pentose phosphate pathway (PPP)-related gene *G6PD* (Figure 2G). Regarding glutamine metabolism-related genes, the presence of glucose decreased the expression of genes encoding glutamine synthetase (*GLUL*), glutamate transporters (*SLC1A2* and *SLC1A3*), and glutamine transporters (*SLC7A5*, *SLC38A1*, and *SLC38A2*) (Figure 2H). A decreased expression of *FASN*, *ACAT1* (acetyl–CoA acetyltransferase 1), *ACADS*, *ACADM*, and *SLC27A1* genes was also observed, encoding, respectively, fatty acid synthase, acetyl–CoA acetyltransferase 1, and short- and medium-chain acyl-CoA dehydrogenases (Figure 2I,J).

U251 cells, exposed to lactate, increased *G6PD* and *SLC2A1* expression, which encodes for the glucose transporter 1, GLUT1 (Figure 2B), as well as the expression of genes related to glutamine and glutamate metabolism, namely *GLS* (glutaminase) and *GLUL*, and *SLC1A3*, *SLC38A2*, and *SLC38A3* genes encoding transporters (Figure 2C), as well as *FASN* expression (Figure 2E). Regarding the expression of genes encoding enzymes involved in ketone body metabolism, lactate decreased the expression of *OXCT1* (3-oxoacid CoA-transferase 1) and *ACAT1* (Figure 2D). U-87MG cells, exposed to lactate, decreased the expression of *HK2* (Figure 2G) and the expression of β-oxidation genes *ACADS* and *ACADM* (Figure 2J), while *G6PD* expression increased, as well as the expression of genes related to glutamine and glutamate metabolism (Figure 2H). 

U251 cells, exposed to glutamine, increased *FASN* expression (Figure 2E). U-87MG cells, exposed to glutamine, decreased the expression of genes related to glutamate and glutamine transport (Figure 2H), as well as to fatty acid β-oxidation (Figure 2J). 

U251 cells, exposed to glutamate, increased *FASN* expression (Figure 2E). U-87MG cells exposed to glutamate decreased the expression of *HK2* (Figure 2G), of genes encoding glutamate transporters (Figure 2H), and of *ACAT1* and *ACADM* (Figure 2J). 

### 3.3. Glucose, Lactate, Glutamine, and Glutamate Bioavailability Impact the Metabolic Profiles of GBM Cell Lines Differently

**Figure 3:**
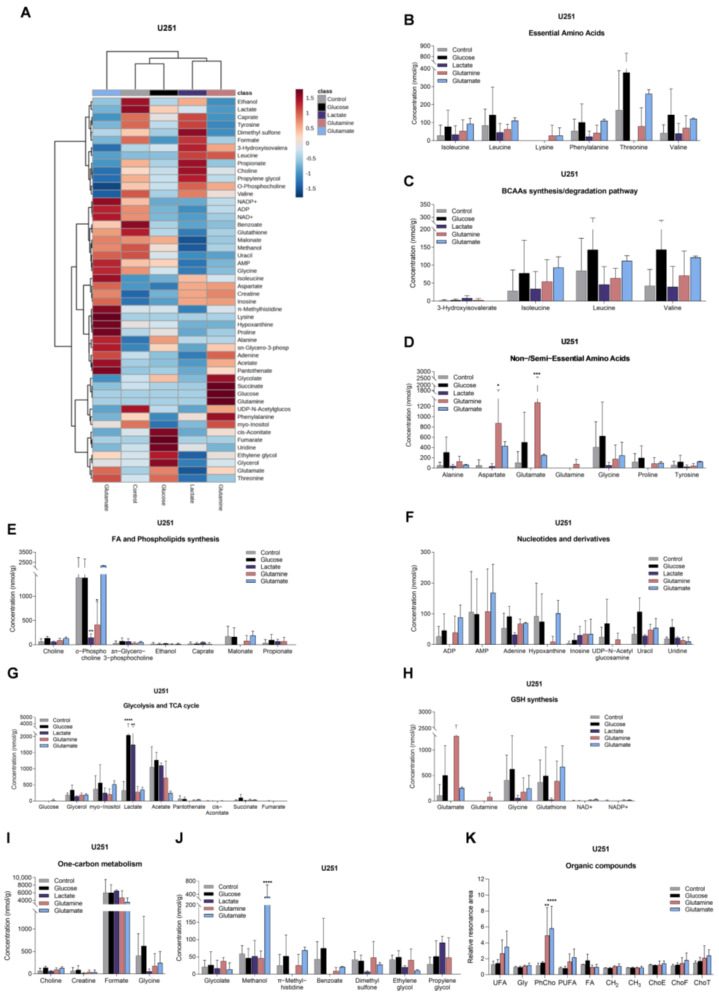
Impact on the levels of metabolites in U251. Metabolite levels obtained from ^1^H-NMR spectra of aqueous phase of U251 cell extracts (heatmap: (**A**)) are divided into several groups: essential amino acids (**B**), BCAA synthesis/degradation pathway (**C**), non/semiessential amino acids (**D**), fatty acid and phospholipid synthesis (**E**), nucleotides and derivatives (**F**), glycolysis and TCA cycle (**G**), glutathione (GSH) synthesis (**H**), one-carbon metabolism (**I**), and other metabolites that did not fit in the previous groups (**J**). Organic compounds obtained from ^1^H-NMR spectra of organic phase of U251 cell extracts (**K**): –CH=CH– (unsaturated FA: UFA), glycerol from triacylglycerol (Gly), phosphatidylcholine (PhCho), –CH=CH–CH_2_–CH=CH– (polyunsaturated FA: PUFA), fatty acids (FA), CH_2_ and CH_3_ from FA elongation, esterified cholesterol (ChoE), free cholesterol (ChoF), and total cholesterol (ChoT). All data are represented as mean ± SD. * *p* < 0.05, ** *p* < 0.01, *** *p* < 0.001, **** *p* < 0.0001.

**Figure 4:**
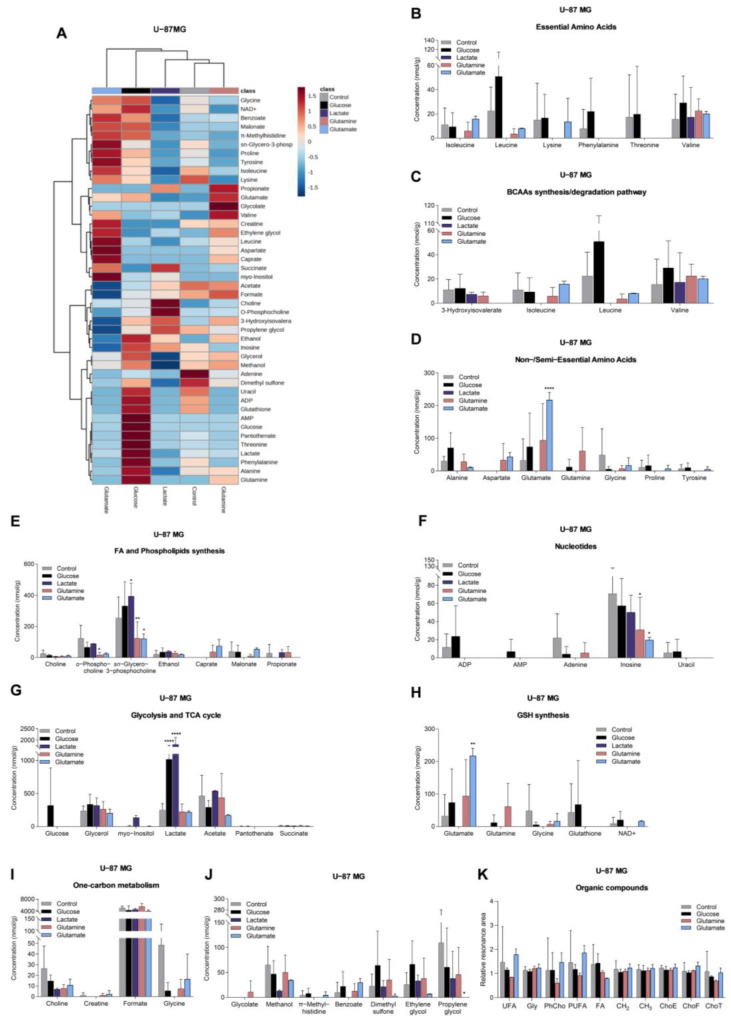
Impact on the levels of metabolites in U-87MG. Metabolite levels obtained from ^1^H-NMR spectra of aqueous phase of U-87MG cell extracts (heatmap: (**A**)) are divided into several groups: essential amino acids (**B**), BCAA synthesis/degradation pathway (**C**), non/semiessential amino acids (**D**), fatty acid and phospholipid synthesis (**E**), nucleotides and derivatives (**F**), glycolysis and TCA cycle (**G**), glutathione (GSH) synthesis (**H**), one-carbon metabolism (**I**), and other metabolites that did not fit in the previous groups (**J**). Organic compounds obtained from ^1^H-NMR spectra of organic phase of U-87MG cell extracts (**K**): –CH=CH– (unsaturated FA: UFA), glycerol from triacylglycerol (Gly), phosphatidylcholine (PhCho), –CH=CH–CH_2_–CH=CH– (polyunsaturated FA: PUFA), fatty acids (FA), CH_2_ and CH_3_ from FA elongation, esterified cholesterol (ChoE), free cholesterol (ChoF), and total cholesterol (ChoT). All data are represented as mean ± SD. * *p* < 0.05, ** *p* < 0.01, **** *p* < 0.0001.

To assess the effect of the different carbon sources (glucose, lactate, glutamine, or glutamate) on the metabolic profile of these two GBM cell lines, the aqueous (Figure S2) and organic phases (Figure S3) of cell extracts were analyzed using ^1^H-NMR spectroscopy. The aqueous metabolic profiles of both cell lines are affected differently by these compounds (Figure 3A and Figure 4A).

U251 cells, exposed to glucose, presented increased levels of lactate, succinate (Figure 3G), uracil, and uridine (Figure 3F), while U-87MG cells increased the levels of glucose, lactate (Figure 4G), leucine, alanine, and glutamine (Figure 4B,D).

In the presence of lactate, U251 cells increased the levels of lactate (Figure 3G) and decreased the concentrations of amino acids (Figure 3B,D,J), malonate, and *o*-phosphocholine (Figure 3E), while U-87MG cells decreased the levels of amino acids (Figure 4B,D), *sn*-glycero-3-phosphocholine, and *myo*-inositol (Figure 4E).

U251 cells, exposed to glutamine, increased levels of several amino acids (Figure 3B,D) and glucose (Figure 3G), while *o*-phosphocholine levels were decreased (Figure 3E). U-87MG cells increased glutamine levels, and decreased levels of other amino acids (Figure 4B,D), *o*-phosphocholine, and *sn*-glycero-3-phosphocholine (Figure 4E).

U251 cells, exposed to glutamate, increased the concentration of amino acids (Figure 3B,D), concomitant with decreased levels of 3-hydroxyisovalerate (Figure 3C), while increasing intracellular glutamate levels in U-87MG cells (Figure 4D), and decreasing the levels of amino acids (Figure 4B), *o*-phosphocholine, and *sn*-glycero-3-phosphocholine (Figure 4E). 

Regarding the spectra of organic phases, there were no significant changes in the lipids identified in the organic fraction among conditions in both cell lines (Figure 3K and Figure 4K). The only exception was phosphatidylcholine in U251 cells (Figure 3K). This lipid constituent increased after glutamine and glutamate exposure.

### 3.4. U251 and U-87MG GBM Cell Lines Are Metabolically Different and upon Exposure to Glucose, Lactate, Glutamine, and Glutamate, the Differences Are Emphasized, Suggesting Different Adaptive Pathways

**Figure 5:**
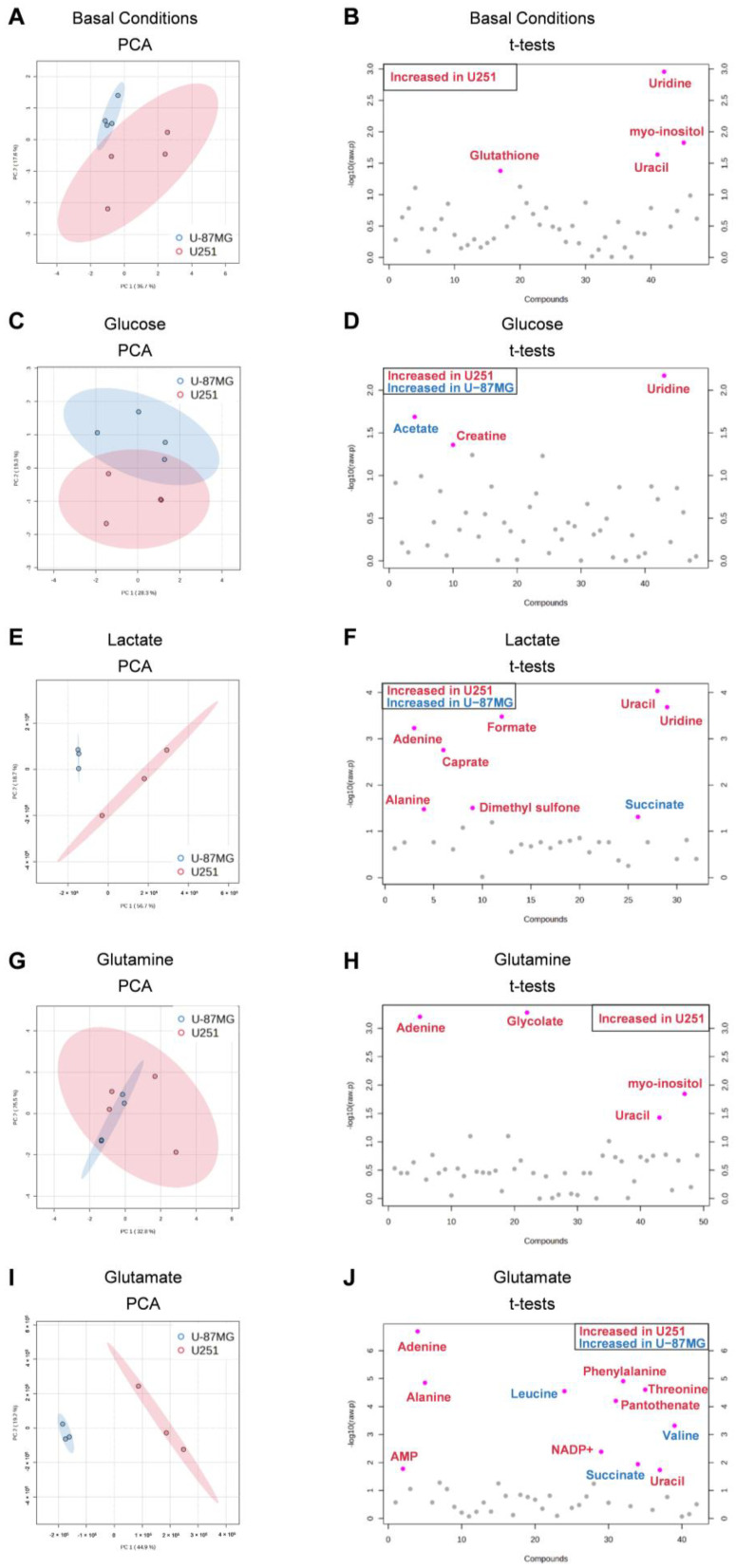
Multivariate and univariate analysis of the impact of key metabolites in both cell lines. Discrimination between U251 (red) and U-87MG (blue) cell lines based on the ^1^H-NMR metabolic profiles of aqueous samples. The number of metabolites identified was 50 and 44 in U251 and U-87MG, respectively. Principal component analysis (PCA) score plots (**A**,**C**,**E**,**G**,**I**) and *t*-test plots (**B**,**D**,**F**,**H**,**J**) of extracts of cells in basal conditions and exposed to glucose, lactate, glutamine, and glutamate, respectively. Metabolites significantly increased in U251 are in red, while metabolites increased in U-87MG are in blue.

To better clarify the difference between each cell line, we performed a multivariate analysis, in which we compared the metabolic profile of each cell line after exposure to each key organic compound. Initially, we observed that at the basal level (cells cultured in control conditions, meaning not exposed to glucose or glutamine) there was a separation of two populations (Figure 5A), with significantly increased glutathione, uracil, uridine, and *myo*-inositol levels in U251 compared to U-87MG (Figure 5C). Upon exposure to glucose, lactate, glutamine, and glutamate, the separation of the two cell lines as independent populations was maintained (Figure 5C,E,G,I) due to differential modulation by the different metabolites. In the case of glutamine (Figure 5G), the clustering of the two cell lines is not so clear on the PCA due to the high variance in the U251 cell line. Upon exposure to glucose, elevated levels of creatine and uridine were found in U251, while U-87MG presented high levels of acetate (Figure 5B). In the presence of lactate, U251 presented higher levels of adenine, alanine, caprate, formate, uracil, and uridine, while U-87MG presented higher succinate levels (Figure 5F). Upon exposure to glutamine, U251 cells presented higher levels of adenine, glycolate, uracil, and *myo*-inositol compared to U-87MG (Figure 5H). In U251, glutamate induced higher levels of AMP, adenine, alanine, NADP+, pantothenate, phenylalanine, threonine, and uracil, while in U-87MG it increased the levels of leucine, succinate, and valine (Figure 5J). 

### 3.5. Glucose, Glutamine, and Glutamate Increase Migration, and Lactate Tends to Decrease Proliferation in GBM Cell Lines

**Figure 6:**
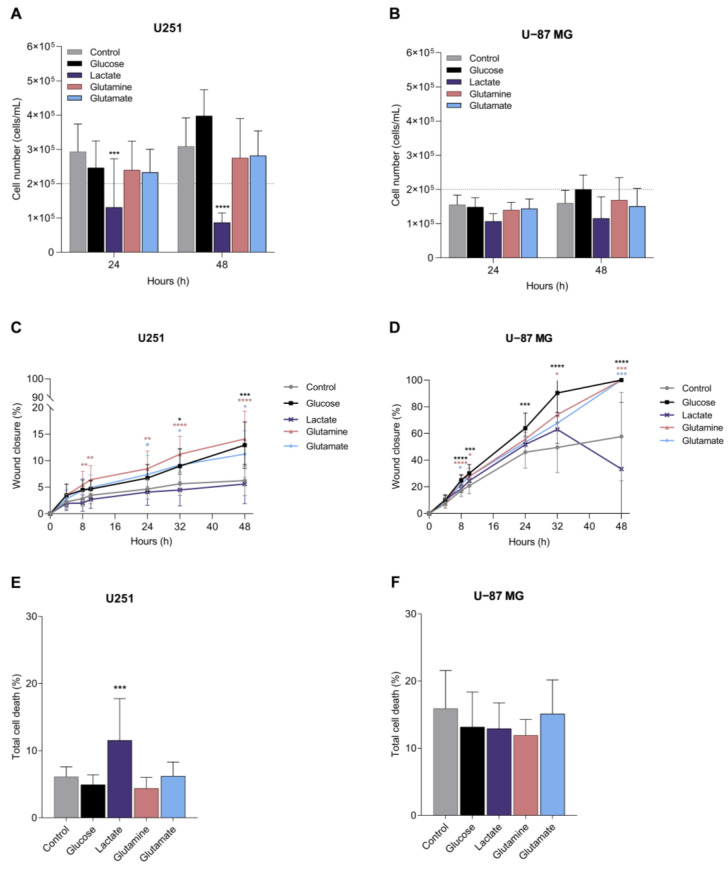
Impact of metabolites in cell features crucial for cancer progression. Features assessed in GBM cell lines: proliferation, migration, and cell death of U251 (**A**,**C**,**E**) and U-87MG (**B**,**D**,**F**). All data are represented as mean ± SD. * *p* < 0.05, ** *p* < 0.01, *** *p* < 0.001, **** *p* < 0.0001.

In order to obtain some information on the way the carbon source and metabolic adaptation can impact disease progression, we assessed the effect of glucose, lactate, glutamine, and glutamate on cell features, namely proliferation, cell death/viability, and migration, which are abilities considered crucial for cancer progression. No significant differences were observed with glucose, glutamine, and glutamate (Figure 6A,B). Only lactate affected cell proliferation by significantly decreasing U251 cell number (Figure 6A) and inducing a tendency to decrease U-87MG cell number (Figure 6B). 

Migration, assessed using a wound healing assay, showed that U251 cells are less migratory than U-87MG cells (Figure 6C,D), and the exposure to glucose, glutamine and glutamate significantly increased U251 and U-87MG cell migration (Figure 6C,D). Lactate did not change the migration rate in U251 cells (Figure 6C) and significantly decreased the wound closure in U-87MG at the end of the assay, between 32 and 48 h of incubation (Figure 6D).

Considering cell death, in general, U251 cells presented lower cell death levels than U-87MG cells, and glucose, glutamine, and glutamate did not affect cell death levels in any cell line (Figure 6E,F). Lactate induced cell death in U251 (Figure 6E), but not in U-87MG (Figure 6F), compared to cells cultured in control conditions. 

## 4. Discussion

During cancer progression, metabolic remodeling occurs, allowing for the adaptation of cancer cells and supporting tumor growth. In this study, we observed that samples from the primary GBM showed an altered transcriptomic profile related to metabolic genes compared to normal tissue (Figure 1 and Supplementary Figure S1). Primary GBM presented an mRNA expression profile compatible with glucose and glutamine dependency and possibly decreased fatty acid synthesis. Increased expression of glycolytic genes in GBM (including *HK2*) [25,26] is correlated with increased immune infiltration and a worse prognosis of GBM [27,28]. Even though glucose is a key metabolite for GBM, in an NMR spectroscopy study, GBM tumors infused with [U-^13^C]glucose had less than 50% glucose-derived acetyl-CoA, which indicates that these tumors use additional substrates for acetyl-CoA synthesis and TCA cycle fueling, providing energy during tumor growth [29]. Moreover, pyruvate carboxylase expression was decreased (Figure 1S), which is needed for glutamine-independent growth of tumor cells, since this enzyme allows these cells to use glucose-derived pyruvate rather than glutamine for anaplerosis [30]. Furthermore, these tumors are also highly dependent on glutamine [2,14], which is the most frequent substitute of glucose in cancer bioenergetics [9,16]. GBMs are characterized by decreased glutamate uptake (Figure 1B) [31] and increased glutamine transporters, as we also observed in Figure 1C [12,13]. In malignant gliomas, GLS2 is commonly downregulated (as we observed in Figure 1E) [32], but GLS1 is the predominant enzyme in the brain, since it is expressed at higher levels than GLS2 [33], allowing for the hydrolysis of glutamine. Even though GBM tumor cells rely on glucose and glutamine, they can use fatty acids (FA) for energy production [34,35]. We observed that *ACADM*, the gene that encodes for medium-chain acyl-coenzyme A dehydrogenase (MCAD), is increased in primary GBM (Figure 1H), suggesting increased fatty acid β-oxidation. In addition to ATP production, fatty acid oxidation contributes with NADH, NADPH, and FADH for cell proliferation [36,37]. Moreover, *FABP7*, the gene that codes for the brain fatty acid-binding protein (B-FABP) [38], is upregulated in brain tumor tissue (Figure 1L) and is associated with a poor prognosis in malignant gliomas due to its effect in inducing cell migration and tumor invasion [39,40]. We also found decreased levels of the genes that encode for FATP2 and FATP4 (Figure 1I,K), while FATP3- and FAT (*CD36*)-encoding genes were increased (Figure 1J,M). FATP2 is less expressed in the brain [41], while FATP4 is highly expressed [42]. This decrease in the primary tumor may suggest that tumor cells do not rely on this FA transporter, since FATP4 knockdown in U-87MG cells do not affect the cell number [43]. Contrarily, FATP3 is weakly expressed in the brain [44,45], being more associated with fatty acid activation instead of uptake [44], but highly expressed in GBM samples [43]. FATP3 knockdown also inhibited the ability of neurospheres to propagate in orthotopic tumor xenografts [46]. Moreover, CD36 translocase is highly expressed in GBM [47] and promotes long-chain FA uptake as well as oxidated lipoproteins [48,49].

We found decreased levels of *FASN* expression in primary GBM compared to normal tissue in the GBM TCGA cohort (Figure 1G), which suggests decreased fatty acid synthesis. This goes against the findings of several groups, in which *FASN* was overexpressed in GBM and was correlated with increased glioma grade [50]. This could be due to the small sample of normal tissue adjacent to tumor found in this cohort with mRNA seq data, which comprised only five samples. Since the expression of *FASN* is highly heterogeneous among these tumors, a larger sample of normal brain tissue would be important for clarifying these results. When we considered the *IDH1* status, increased *FASN* expression was observed in *IDH1*-mutated tumors, compared to *IDH1* wt (Figure 1T). In fact, *IDH1* mutations lead to increased BCAA levels [51], since α-ketoglutarate is not available for BCAA transamination once it is converted into the oncometabolite 2-hydroxyglutarate (D2-HG) by IDH1 mutation. Moreover, the branched-chain amino acid transaminase (BCAT1/2) is inhibited by D2-HG [52] in IDH1 mutant tumors, and BCAT promoter methylation also occurs, which leads to BCAT epigenetic silencing [53]. When BCAA metabolism is suppressed, it leads to decreased fatty acid levels, since BCAAs can be a carbon source for acetyl-CoA and, consequently, fatty acid biosynthesis [54,55].

The impact of the availability of different organic compounds as carbon sources on cell malignancy features was evaluated in GBM cell lines, presenting different phenotypes. The U251 cell line is more proliferative than that of U-87MG, which is a more migratory cell line (Figure 6). These features were disclosed in vitro but also in vivo since a study comparing U251 and U-87MG xenograft tumor models showed that U251 proliferated more, creating more cell nests and secondary tumors whereas the U-87MG model presented faster infiltrative tumor growth, with larger tumor volume and decreased overall survival [56]. Another study also showed that U-87MG migrates and invades more than U251 [57]. In addition to these phenotypic differences [58], these cell lines also presented differences at the metabolic level, with U251 having increased antioxidant and pyrimidine levels than U-87MG (Figure 5B). Proteomic and RT-qPCR analyses showed differences between these two cell lines, mainly in nicotinamide nucleotide metabolic process regulation, RNA splicing, glycolysis, and purine metabolism [57]. 

**Figure 7:**
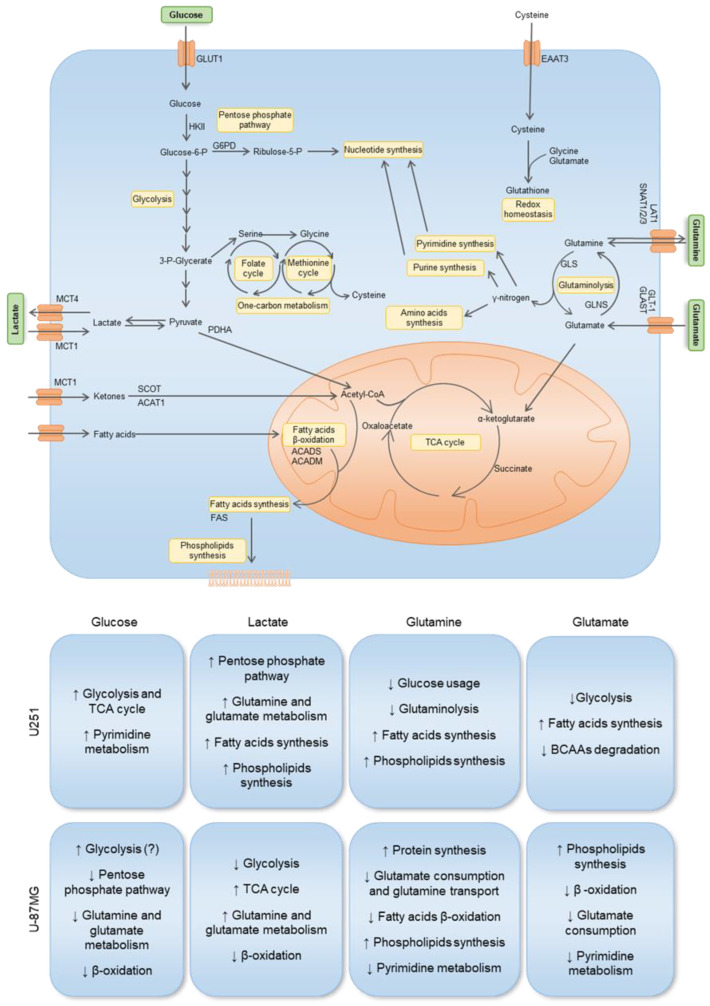
Impact of key metabolites in the gene expression and metabolic profiles of two GBM cell lines: U251 and U-87MG. The uptake of glucose occurs through the GLUT1 transporter. Hexokinase 2 (HKII) is the first rate-limiting enzyme in the glycolytic pathway. Glucose-6-phosphate can be diverted to the pentose phosphate pathway (PPP) through glucose-6-phosphate dehydrogenase (G6PD), contributing to nucleotide synthesis. Glycolysis-derived pyruvate can be converted into lactate, which can be exported and imported by the monocarboxylate transporters MCT4 and MCT1, respectively. Pyruvate oxidation catalyzed by pyruvate dehydrogenase (PDHA) creates acetyl-CoA, which can enter the TCA cycle. Acetyl-CoA can also be derived from ketone body oxidation, which occurs through 3-oxoacid CoA-transferase 1 (SCOT) and acetyl-CoA acetyltransferase 1 (ACAT1). Acetyl-CoA is also a product of fatty acid β-oxidation, where ACADS and ACADM are involved in the breakdown of small- and medium-chain fatty acids, respectively. Acetyl-CoA can be deviated to supply fatty acid synthesis, which occurs through fatty acid synthase (FAS), contributing to phospholipid synthesis. Glutamine is transported through LAT1, SNAT1, SNAT2, and SNAT3, and is hydrolyzed by glutaminase (GLS) into glutamate, generating nitrogen, which is then used for amino acid, purine, and pyrimidine synthesis. Glutamate can also be imported through GLT-1 and GLAST, then being converted into α-ketoglutarate, entering the TCA cycle, or into glutamine, through glutamine synthase (GLNS). Glutamine-derived α-ketoglutarate entering the TCA cycle and contributing to fatty acid metabolism reinforces the importance of glutamine, which is not only used for biomass production, but also for energetic purposes. Glutamine-derived glutamate is used as a nitrogen source in the synthesis of serine from glucose-derived 3-phosphoglycerate. Serine can be converted into glycine, supplying one-carbon metabolism (folate and methionine cycle), from which cysteine is synthesized through the transsulfuration pathway. Glutamate and cystine, alongside glycine, constitute the three components of glutathione, the cell’s most important reactive oxygen species (ROS) scavenger and detoxifying agent. Arrows mean decrease (↓), and increase (↑). Unknown or uncertain course is (?) signed.

In U251 cells, gene expression and metabolic profiling using ^1^H-NMR indicate that glucose exposure increased glycolysis, TCA cycle, and pyrimidine synthesis, while in U-87MG, glycolysis is also increased, but the PPP is decreased (decreased *G6PD*), as well as the glutamine and glutamate metabolism and fatty acid metabolism (decreased *SLC27A1*, *ACADS* and *ACADM*) (Figure 7). Exposure to glucose also induced migration of both cell lines (Figure 6). This suggests that, in U251, glucose induces more energy production, leading to increased migration, while U-87MG presents a higher reliance on glucose with concomitant decrease in FA uptake and degradation. Accordingly, both cell lines presented increased lactate levels after glucose exposure, indicating the occurrence of glycolysis. Actually, astrocytes have a specific glucose-dependent metabolic profile that consists of the production of lactate to support the energy demand of neurons [59]. In fact, we observed elevated levels of *LDHA* in the primary GBM TCGA cohort (Figure 1K) and increased lactate levels in both cell lines using ^1^H-NMR (Figure 3G and Figure 4G). Therefore, GBM cells may retain some of the metabolic characteristics of the cells of origin, astrocytes, which constitute a benefit in the organ microenvironment. Moreover, higher lactate levels are correlated with higher glioma grade [60], since tumor growth is promoted by lactate uptake and consumption, dependent on MCT1 [61]. Additionally, in U251, there is a significant decrease in malonate and *o*-phosphocholine, which may indicate increased fatty acid synthesis and consequent phospholipid synthesis [62,63].

Regarding lactate exposure, it affected several metabolic pathways in U251: PPP, glutamine and glutamate metabolism, fatty acid synthesis, and phospholipid synthesis (Figure 7). It is important to emphasize that the medium in which these cells were cultured did had neither glucose nor glutamine, contrary to other papers that evaluated the effect of lactate exposure in GBM cells in complete media [61,64]. Therefore, our results indicate that lactate, in part, substitutes glucose, as it is a gluconeogenic substrate [65] and glycolysis/gluconeogenesis intermediate glucose-6-phosphate can be deviated into PPP and amino acid synthesis, while lactate can also be converted into pyruvate and supply the TCA cycle and fatty acid synthesis as an acetyl-CoA source [66]. An increase in the glucose transporter GLUT1 (*SLC2A1*) also suggests an attempt to increase glucose uptake (Figure 2B). Regarding ketone body metabolism, the expression of genes encoding enzymes and transporters involved was decreased (Figure 2D), possibly because ketone bodies use the same transporter as lactate to enter cells [67]. 

In U-87MG exposed to lactate, there was a decrease in glycolysis but an increase in TCA cycle, which can indicate that lactate is being converted into pyruvate and fueling the TCA cycle with carbons through acetyl-CoA [68,69]. There is also an increase in glutamine and glutamate metabolism and a decrease in fatty acid β-oxidation. This indicates that the U-87MG cell line also adapts easily to the absence of glucose and glutamine, being able to use lactate as a carbon source, which is reflected in the unaffected cell proliferation after lactate exposure (Figure 6). However, several amino acids decreased after lactate exposure (Figure 4B,D), which suggests increased protein synthesis/amino acid consumption or decreased amino acid synthesis, the latter being the most feasible hypothesis, since these cells did not have glutamine in the culture medium, which provides the nitrogen needed for amino acids synthesis [70,71]. Moreover, glutamine is the main source of glutamate, which constitutes a metabolic hub, having a core role in supplying different metabolic pathways [72]; thus, it makes sense that the consumption of amino acids is decreased in the absence of glutamine.

Glutamine exposure decreased glutaminolysis and the use of glucose by U251, while increasing fatty acid and phospholipid synthesis (Figure 6). Regarding U-87MG, there was a decrease in glutamate consumption and glutamine transport, since glutamine also provides the nitrogen needed for amino acid synthesis. Fatty acid β-oxidation was decreased, while phospholipid synthesis was increased (Figure 7), which are elements of cell membranes and essential to supporting proliferation and tumor growth [73]. Pyrimidine metabolism was also decreased.

Glutamate exposure increased fatty acid synthesis while decreasing BCAA degradation and glycolysis in U251 (Figure 7). In U-87MG, there was decreased glutamate consumption, reduced pyrimidine metabolism and β-oxidation, while phospholipid synthesis increased. This suggests that, in both cell lines, glutamate is channeled into fatty acid and phospholipid synthesis [74,75].

Regarding the synthesis of FAs in GBM cell lines, we saw that it may increase since the expression of FASN was upregulated in the presence of glutamine. It is known that glutamine can be used to sustain de novo lipogenesis [76], which might indicate that glutamine is important to meet the metabolic requirements of GBM cells for FA synthesis to occur. These results, however, do not fully translate into the organic fraction of cells exposed to different conditions (Figure 3K and Figure 4K). It was observed that the dynamics of lipids stay similar to the control condition, the only exception being phosphatidylcholine in the U251 cell line (Figure 3K), which increased, separately, in the presence of glutamine and glutamate. When glucose was added, relative phosphatidylcholine levels were similar to control levels. Phosphatidylcholine is an important structural lipid, being one of the most abundant phospholipids in the cell membrane [77]. Furthermore, *o*-phosphocholine is a precursor of phosphatidylcholine [78], and U251 presented decreased levels of *o*-phosphocholine upon exposure to glutamine (Figure 3E), which fits with an increased flow of phosphatidylcholine production. In the case of U-87MG cells (Figure 2J), the expression of genes related to fatty acid oxidation and synthesis decreased in most conditions, with no impact on lipid dynamics in organic phases (Figure 4K). The development of a more targeted approach to lipid profiling is necessary to better understand the effects of nutrient availability on the metabolism of lipids. Hence, we observed that each carbon source induced different responses in each cell line and that cells can adapt to it, increasing a differential flux of metabolic pathways to maintain cell proliferation and migration (Figure 7). 

Thus, assessing the impact of each metabolite is important for a better understanding of GBM biology. Lactate is pointed out as a valuable energetic and biomass source and besides the two cell lines present different adaptive courses to lactate, they are both able to metabolize it and adapt the expression and metabolic profiles to lactate availability. This shows an opportunity to use lactate-related metabolic players as markers and targets in GBM, MCT1 and MCT4 being the main candidates since they are the most explored elements in cancer context [79]. In this paper, we also highlighted the fatty acid metabolism and the glutamine/glutamate metabolism as putative targets of anti-GBM therapies. As mentioned in a previous review paper from our group [14], several strategies are being developed to target the glutamine metabolism, including transporter inhibitors, glutaminase blocking, and GLT-1 overexpression, among others. Regarding fatty acid metabolism, pharmacological inhibition of SCD and FADS2 in GBM cells induced palmitate accumulation, which, in combination with TMZ, increased cell death [80]. Moreover, Duman and colleagues showed that blocking fatty acid oxidation through acyl-CoA binding protein induced immobility [81], which is very relevant since invasion is one of the major characteristics of these tumors. Additionally, targeting fatty acid oxidation reduces energy production and cell proliferation [37]. In a study by Pike and colleagues, the GBM cell line SF188 was exposed to etomoxir (a carnitine palmitoyltransferase 1 inhibitor), which inhibits fatty acid β-oxidation, and the levels of ATP, NADPH, and glutathione (GSH) decreased, followed by decreased viability [82].

Unfortunately, these strategies present severe limitations, mainly due to the high heterogeneity of these tumors, and the major anatomical and molecular obstacle of treating brain tumors, the blood–brain barrier (BBB), making treatment very challenging with often poor success. The reduction in the bioavailability of preferential substrates for a tumor can be a way of interfering with metabolic adaptation and controlling tumor growth. Besides some limitations, such as the low number of cell lines evaluated, though tested in biological triplicates, we believe our study is a contribution to better understanding which core metabolic pathways act on GBM. Therefore, new strategies must be tested in order to reduce the access of certain organic compounds to the brain and block the metabolic adaptation of GBM cells. On the other hand, taking advantage of the players expressed by tumors as mediators of drug delivery systems could be an opportunity to improve the clinical management of GBM, ensuring more specific delivery and protecting normal cells from adverse effects.

## 5. Conclusions

In the future, metabolic profiling could contribute to better diagnosis and follow-up of the metabolic drift accompanying disease progression and therapy response in GBM. According to the metabolome, we will be able to identify which pathways are overactivated in order to allow for the use of metabolism-based therapies to target key players, keeping in mind that the treatment must be adjusted according to the disease’s progression and to tumor cells’ metabolic adaptation, which can be mapped using metabolome profiling.

## Figures and Tables

**Table 1:** List of primers used in RT-qPCR assays.

	Gene	Protein	Primer Forward	Primer Reverse
Glucose-dependent pathways	*HK2*	HKII	GGAGAGGGGACTTTGATATCG	CGCATCTCTTCCATGTAGCAG
	*G6PD*	G6PD	GGCAACAGATACAAGAACGTGAAG	GCAGAAGACGTCCAGGATGAG
	*PDHA1*	PDHA	GCTAACCAGGGCCAGATATTC	CTTGTAGTAATCAGTGCTGGC
	*SLC2A1*	GLUT1	CACGGCCTTCACTGTCGTG	GGACATCCAGGGTAGCTGC
	*SLC16A1*	MCT1	GCTGGGCAGTGGTAATTGGA	CAGTAATTGATTTGGGAAATGCA
	*SLC16A4*	MCT4	CACAAGTTCTCCAGTGCCATTG	CGCATCCAGGAGTTTGCCTC
Glutamine-dependent pathways	*GLS*	GLS1	CTTCTACTTCCAGCTGTGCTC	CACCAGTAATTGGGCAGAAACC
	*GLUL*	GLNS	GAATGGTCTGAAGTACATCGAGG	GTTAGACGTCGGGCATTGTC
	*SLC1A2*	GLT-1	GGGATGAACGTCTTAGGTCTG	GGGGAGAGTACCACATGATC
	*SLC1A3*	GLAST	CACCGCTGTCATTGTGGGTAC	CCGCCATTCCTGTGACAAG
	*SLC7A5*	LAT1	CATCCTCCAGGCTCTTCTTC	CGTCATCACACACGTGAACAC
	*SLC38A1*	SNAT1	CATTCTATGACAACGTGCAGTCC	CAGCAACAATGACAGCCAGC
	*SLC38A2*	SNAT2	CTGAGCAATGCGATTGTGGG	CTCCTTCATTGGCAGTCTTC
	*SLC38A3*	SNAT3	CACAGACAGCATACACCATCC	GACAGGTTGGAGATGTGCTGC
Acetoacetate metabolism	*OXCT1*	SCOT	GGCCGCTCTTGAGTTTGAGG	CGTGGATATGGACCCAAACC
	*ACAT1*	ACAT1	GTATTGGGTGCAGGCTTACC	CATTGGACATGCTCTCCATCC
Lipid metabolism	*ACADS*	SCAD	CCCTCGATTGTGCTGTGAAC	GCCAACTTGAACTGGATGACC
	*ACADM*	MCAD	GCTACTTGTAGAGCACCAAGC	CCAAGCTGCTCTCTGGTAAC
	*FASN*	FAS	GCTCGGCATGGCTATCTTC	GGAACACCGTGCACTTGAGG
Housekeeping	*HPRT*	HPRT	TGACACTGGCAAAACAATGCA	GGTCGTTTTTCACCAGCAAGCT

## Data Availability

Not applicable.

## Supplementary Materials

The following supporting information can be downloaded at: https://www.mdpi.com/article/10.3390/biomedicines11072041/s1, Table S1. Results of Shapiro–Wilk normality test. To access if normal tissue, primary and recurrent tumor samples presented a normal distribution, the Shapiro–Wilk test was performed for all the mRNAseq data related to each gene evaluated in the TCGA GBM cohort, due to the small sample size of normal and recurrent tumor samples (*n* < 50). All data were analyzed using GraphPad Prism. Figure S1. Gene expression profile between glioblastoma (GBM) (primary and recurrent tumor) and normal tissue in GBM TCGA cohort. The expression of genes that code for enzymes or transporters involved in several pathways was assessed. Genes related to glutamine metabolism: *GLUL* (A), *SLC1A3* (B), *SLC7A5* (C), *SLC7A8* (D), *SLC7A11* (E), *SLC38A1* (F), *SLC38A2* (G), and *SLC38A3* (H). Genes related to glucose, pyruvate, and lactate metabolism: *SLC2A1* (I), *G6PD* (J), and *LDHB* (K). Genes related to cysteine metabolism: *SLC7A11* (E), *CTH* (L), *MPST* (M), and *CBS* (N). Genes related to fatty acid metabolism: *ACADS* (O), *ACADL* (P), *ACADVL* (Q), *SLC27A1* (R), *SLC27A5* (S), *SLC27A6* (T), *FABP3* (U), and *FABP5* (V). All data are represented as mean ± SD. * *p* < 0.05, ** *p* < 0.01, *** *p* < 0.001. Figure S2. Representative ^1^H-NMR spectra of aqueous phases of GBM cell lines. Representative ^1^H-NMR spectra of aqueous phase of U251 cell line under baseline conditions (A), exposed to glucose (B), lactate (C), glutamine (D), and glutamate (E). Representative ^1^H-NMR spectra of aqueous phase of U-87MG cell line in baseline conditions (F), exposed to glucose (G), lactate (H), glutamine (I), and glutamate (J). Increased compounds are in blue and decreased compounds in red. Figure S3. Representative ^1^H-NMR spectra of organic phases of GBM cell lines. Representative ^1^H-NMR spectra of organic phase of U251 cell line under baseline conditions (A), exposed to glucose (B), lactate (C), glutamine (D), and glutamate (E). Representative ^1^H-NMR spectra of organic phase of U-87MG cell line in baseline conditions (F), exposed to glucose (G), lactate (H), glutamine (I), and glutamate (J). Increased compounds are in blue.
