# Supplementary material for: Metabolic Profiles Point Out Metabolic Pathways Pivotal in Two Glioblastoma (GBM) Cell Lines, U251 and U-87MG
Source: Biomedicines. 2023 Jul 20;11(7):2041. doi: 10.3390/biomedicines11072041 (PMC10377067; doi:10.3390/biomedicines11072041)
Supplement: Supplementary file 1 [file biomedicines-11-02041-s001.zip › biomedicines-2493662-supplementary.pdf]

Supplementary

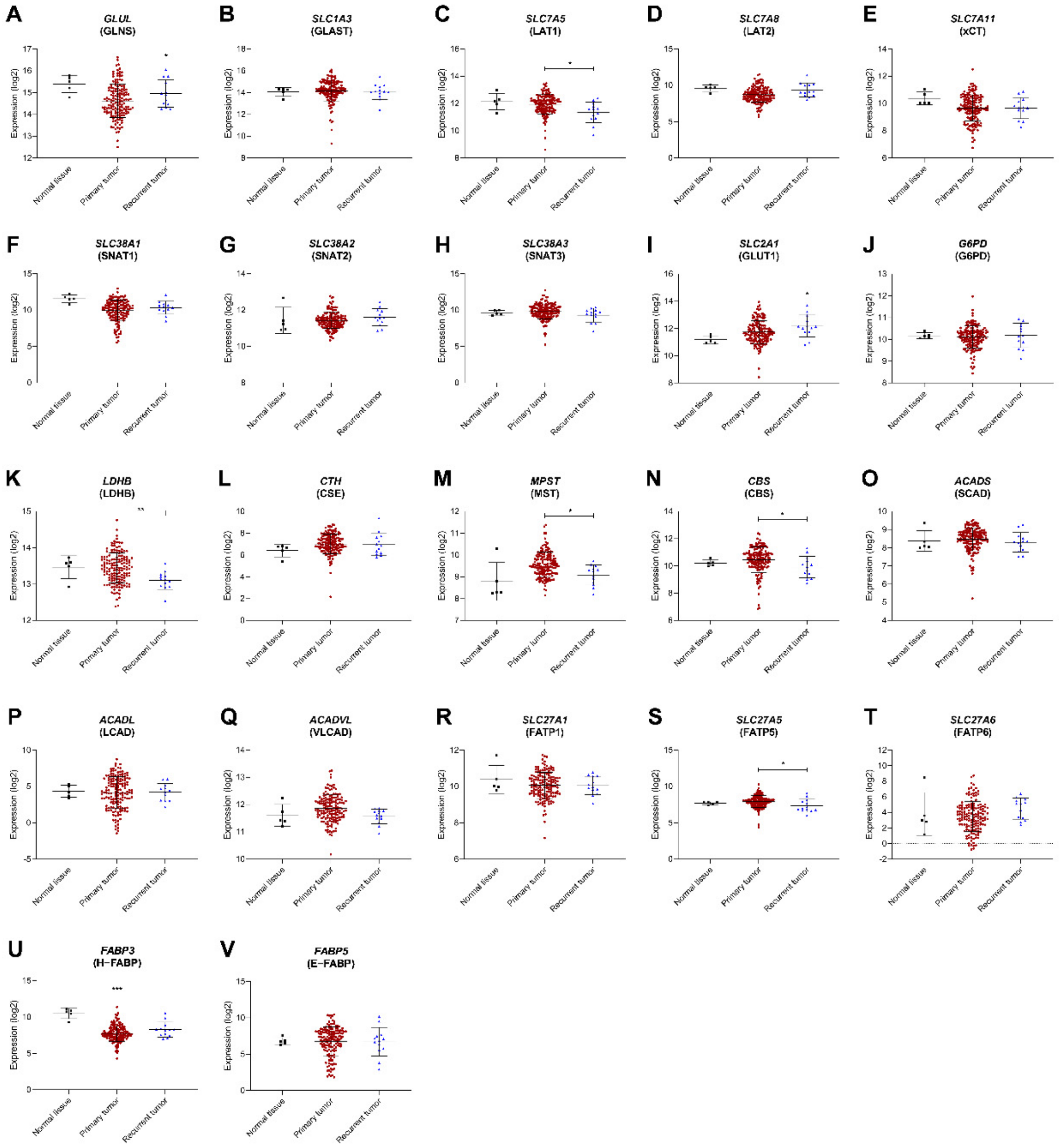

Figure S1. Gene expression profile between glioblastoma (GBM) (primary and recurrent tumor) and nor-mal tissue in GBM TCGA cohort. The expression of genes that code for enzymes or transporters involved in several pathways was assessed. Genes related to glutamine metabolism: GLUL (A), SLC1A3 (B), SLC7A5 (C), SLC7A8 (D), SLC7A11 (E), SLC38A1 (F), SLC38A2 (G) and SLC38A3 (H). Genes related to glucose, pyruvate and lactate metabolism: SLC2A1 (I), G6PD (J), and LDHB (K). Genes related to cysteine metabolism: SLC7A11 (E), CTH (L), MPST (M), and CBS (N). Genes re-lated to fatty acid metabolism: ACADS (O), ACADL (P), ACADVL (Q), SLC27A1 (R), SLC27A5 (S), SLC27A6 (T), FABP3 (U), and FABP5 (V). All data is represented as mean ± SD. *p<0.05, **p<0.01, ***p<0.001.

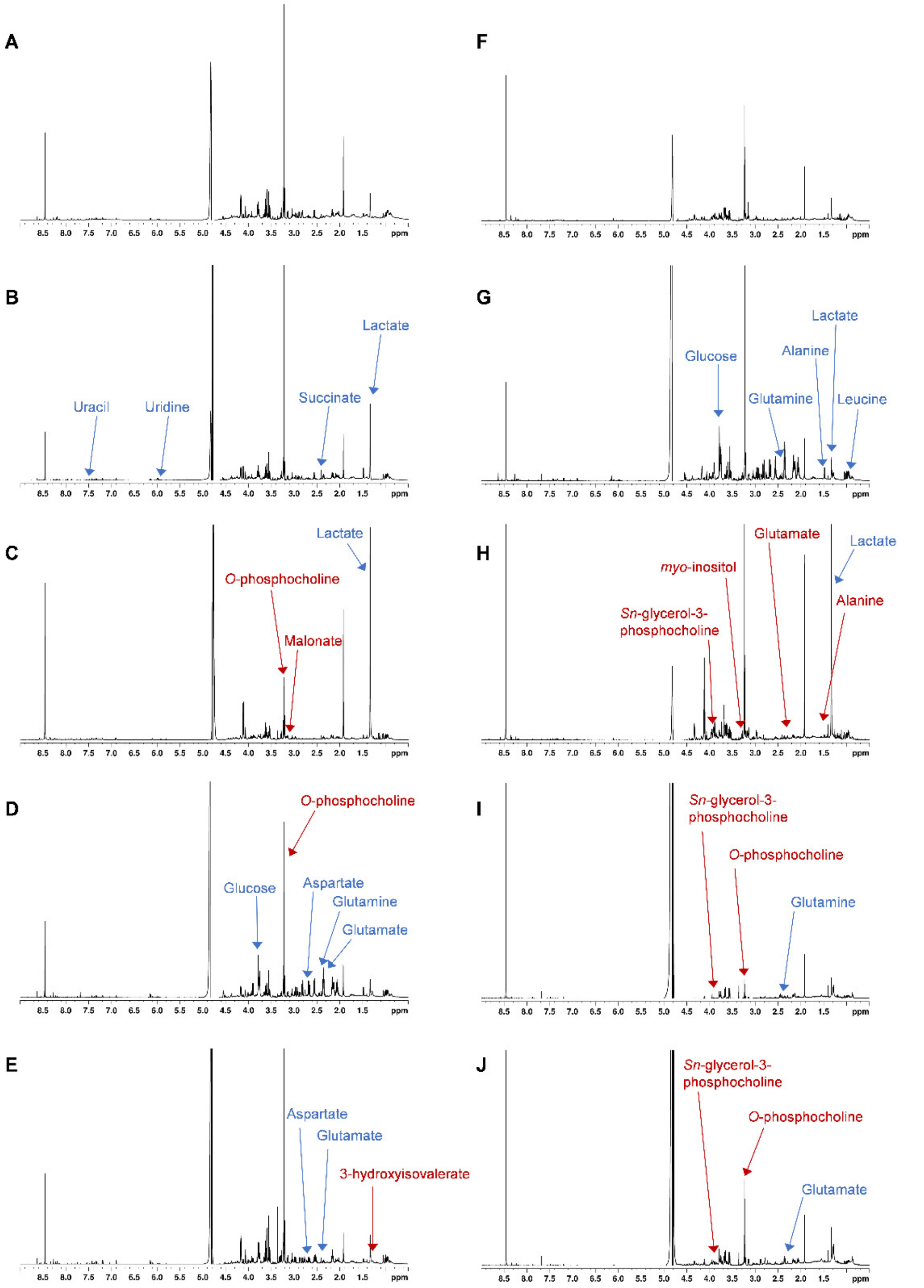

Figure S2. Representative 1H-NMR spectra of aqueous phases of GBM cell lines. Representative 1H-NMR spectra of aqueous phase of U251 cell line in baseline conditions (A), exposed to glucose (B), lactate (C), glutamine (D), and glutamate (E). Representative 1H-NMR spectra of aqueous phase of U-87MG cell line in baseline conditions (F), exposed to glucose (G), lactate (H), gluta-mine (I), and glutamate (J). Increased compounds are in blue and the decreased compounds in red.

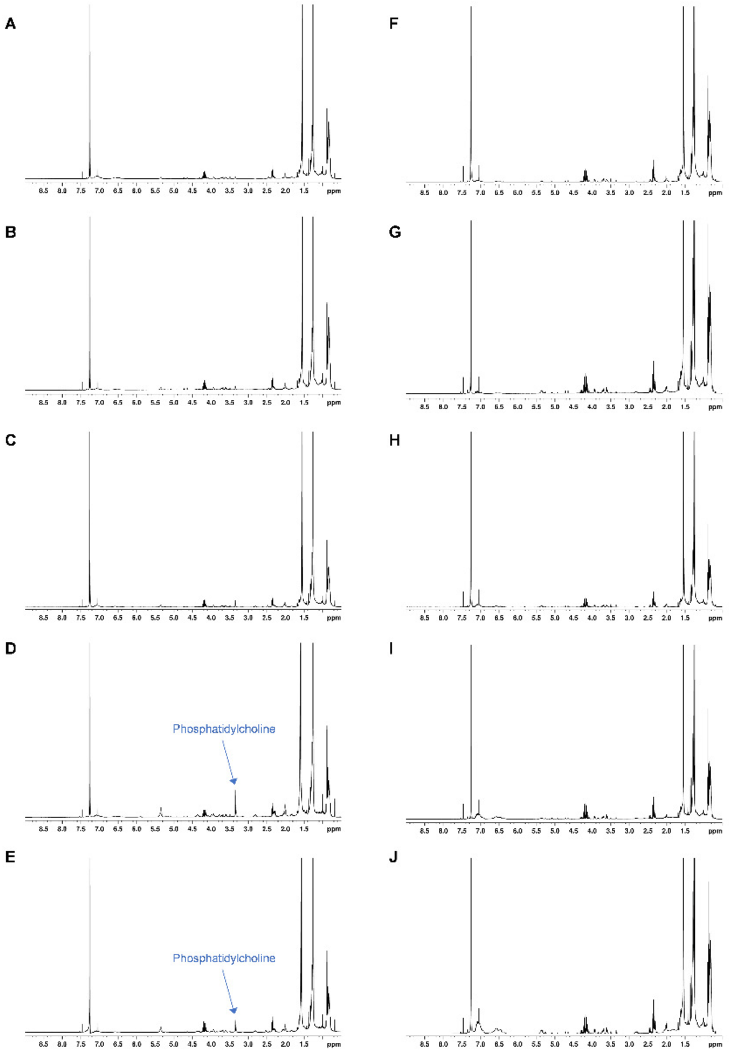

Figure S3. Representative 1H-NMR spectra of organic phases of GBM cell lines. Representative 1H-NMR spectra of organic phase of U251 cell line in baseline conditions (A), exposed to glucose (B), lactate (C), glutamine (D), and glutamate (E). Representative 1H-NMR spectra of organic phase of U-87MG cell line in baseline conditions (F), exposed to glucose (G), lactate (H), glutamine (I), and glutamate (J). Increased compounds are in blue.

Table S1. Results of Normality test Shapiro-Wilk. To access if normal tissue, primary and recurrent tumor samples presented a normal distribution, Shapiro-Wilk test was performed for all the mRNAseq data related to each gene evaluated on the TCGA GBM cohort, due to the small sam-ple size of normal and recurrent tumor samples (n<50). All data was analyzed by GraphPad Prism.

	**Shapiro-Wilk test**		
	**Passed Normality test (alpha=0.05)?**		
	**Normal**	**Primary tumor**	**Recurrent tumor**
**Gene**	**n=5**	**n=153**	**n=13**
GLS	No	No	Yes
GLS2	No	Yes	Yes

GLUL	Yes	Yes	Yes
SLC1A1	Yes	Yes	Yes
SLC1A2	No	No	Yes
SLC1A3	No	No	Yes
SLC1A5	Yes	No	Yes
SLC7A5	Yes	No	Yes
SLC7A8	Yes	Yes	Yes
SLC7A11	Yes	Yes	Yes
SLC38A1	Yes	No	Yes
SLC38A2	Yes	No	Yes
SLC38A3	Yes	No	Yes
SLC2A1	Yes	No	Yes
HK2	Yes	Yes	Yes
G6PD	Yes	No	Yes
PDHA1	Yes	No	Yes
PC	Yes	Yes	Yes
SLC16A1	Yes	Yes	Yes
SLC16A4	Yes	No	Yes
LDHA	Yes	No	No
LDHB	Yes	Yes	Yes
CTH	No	No	Yes
MPST	No	Yes	Yes
CBS	Yes	No	Yes
ACADS	No	No	Yes
ACADM	Yes	No	Yes
ACADL	Yes	No	Yes
ACADVL	Yes	Yes	Yes
SLC27A1	Yes	Yes	Yes
SLC27A2	Yes	No	Yes
SLC27A3	Yes	No	Yes
SLC27A4	Yes	Yes	Yes
SLC27A5	Yes	No	Yes
SLC27A6	Yes	Yes	Yes
CD36	Yes	Yes	Yes
FABP3	Yes	Yes	Yes
FABP5	Yes	No	Yes
FABP7	Yes	No	Yes
FASN	Yes	No	Yes
